# Change of Peri-mitral Atrial Flutter into Single-loop Biatrial Flutter During Ablation

**DOI:** 10.19102/icrm.2023.14044

**Published:** 2023-04-15

**Authors:** Ele Wu, Dominika Zoltowska, Jialin Su

**Affiliations:** ^1^Division of Cardiology, University of Florida College of Medicine, Jacksonville, FL, USA; ^2^Division of Cardiology, Berkshire Medical Center, Pittsfield, MA, USA

**Keywords:** Ablation, atypical atrial flutter, biatrial flutter, high-resolution mapping, peri-mitral atrial flutter

## Abstract

Anterior line ablation for peri-mitral atrial flutter (AFL) is associated with biatrial flutter due to disruption of the electrical conduction in the left atrial septum. An AFL case with valvular disease, cardiac surgery, and prior ablation was confirmed to be counterclockwise peri-mitral flutter with isthmus on the left atrial septum. Ablation on the septum of the left atrium (LA) targeting the isthmus prolonged the tachycardia cycle length (TCL) from 266 to 286 ms. Left atrial mapping during AFL with a TCL of 286 ms showed that the activation remained peri-mitral counterclockwise, but there was interruption of the local activation time (LAT) sequence. Combined mapping of the LA and the right atrium (RA) showed a counterclockwise single-loop biatrial flutter, involving the whole LA and the RA septum, with Bachmann’s bundle and the posteroinferior septum being the interatrial connections. The AFL was terminated by ablation at the right superior cavoatrial junction. RA mapping should be considered if there is prolongation of TCL but without termination of the peri-mitral AFL, and if there is interruption of the continuity of the LAT sequence during AFL with a longer TCL. The biatrial flutter can be terminated by ablation targeting the interatrial connections.

## Case presentation

**Figure 1:**
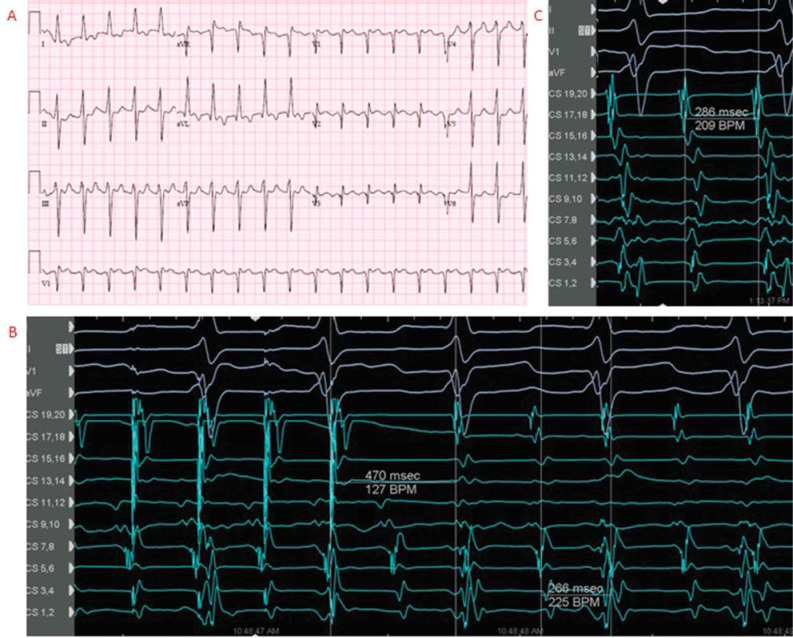
Twelve-lead electrocardiogram **(A)**, entrainment from the right atrial free wall **(B)**, and prolongation of the tachycardia cycle length during ablation **(C)**. A duodecapolar catheter was advanced into the right atrium, with distal catheter in the coronary sinus (CS). CS_1,2_ to CS_9,10_ were located in the CS and CS_11,12_ to CS_19,20_ were located in the RA around the tricuspid annulus, respectively.

A 58-year-old man without known chronic disease presented in a 2:1 atrial flutter (AFL) **(Figure 1A)** without symptoms at his primary care physician’s office. An echo showed severe left ventricular (LV) systolic dysfunction with severe aortic insufficiency, moderate mitral regurgitation, and ascending aortic aneurysm, for which the Bentall procedure was performed with a mechanical valve conduit and mitral valve annuloplasty. Placement of a left atrial (LA) appendage (LAA) clip and the Maze procedure were also completed during the surgery. The coronary angiogram was normal, and the AFL converted to sinus rhythm spontaneously before the operation. After surgery, the patient underwent empiric cavotricuspid isthmus (CTI) ablation during sinus rhythm and had recurrent AFL. He was discharged on amiodarone after direct current cardioversion; however, he went into AFL again despite taking amiodarone. The LV ejection fraction (LVEF) remained severely decreased after the surgery.

The presenting rhythm in the electrophysiology laboratory was AFL with a tachycardia cycle length (TCL) of 266 ms. Coronary sinus (CS) activation was from proximal to distal. Entrainment from the proximal CS showed the post-pacing interval (PPI)-TCL to be +30 ms. Entrainment from the RA free wall showed the PPI-TCL to be +204 ms **(Figure 1B)**, consistent with left AFL.

**Figure 2:**
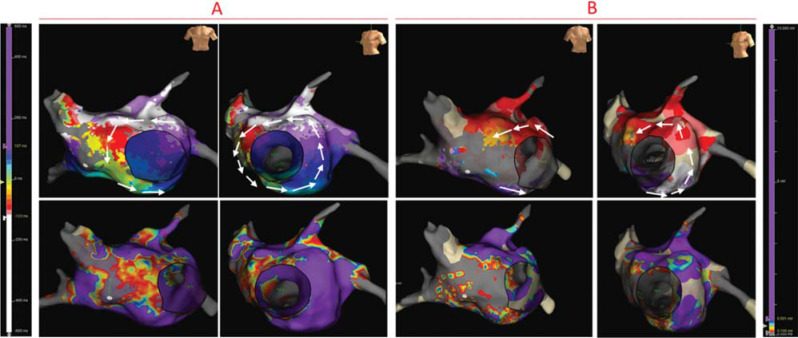
Left atrial local activation time and bipolar voltage mapping during atrial flutter with a tachycardia cycle length of 266 ms **(A)** or 286 ms **(B)**. The activation sequence is indicated by white arrows.

High-resolution 3-dimensional (3D) electroanatomic mapping of the LA and pulmonary veins (PVs) was obtained (Advisor™ HD Grid Mapping Catheter and EnSite Precision™ 3D mapping system; Abbott, Chicago, IL, USA). Activation in the LA was counterclockwise around the mitral annulus (MA). Bipolar voltage mapping showed normal voltage (>0.5 mV) except for a low-voltage area in the septum **(Figure 2A)**. Entrainment around the MA at 2, 7, and 9 o’clock all showed the PPI-TCL to be <+20 to +30 ms. The 3D mapping and entrainment confirmed a diagnosis of counterclockwise peri-mitral AFL **(Figure 2A)**. Anterior line ablation from the right superior PV to the MA was performed along the low-potential region on the LA septum, which harbored the isthmus of the AFL with conduction velocity deceleration, as shown in the propagation map. The TCL was prolonged from 266 to 286 ms during ablation lesion delivery but did not terminate **(Figure 1C)**. The right PVs were then isolated by ablation without alteration or termination of the AFL.

**Figure 3:**
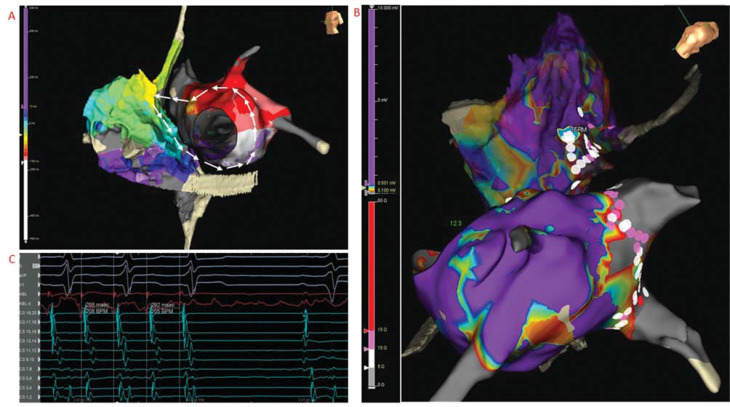
Local activation time mapping of the single-loop biatrial flutter **(A)**, ablation lesions **(B)**, and termination of the atrial flutter during ablation **(C)**. The activation sequence is indicated by white arrows.

Additional 3D mapping of the LA was performed during AFL with a TCL of 286 ms. It was noted that there was interruption of the activation sequence continuity (3 colors [yellow, green, and blue] were “missing” on the local activation time [LAT] mapping) **(Figure 2B)**. The RA was mapped, and the earliest activation was below the superior vena cava (SVC) and at the superior cavoatrial junction. The propagation from the right superior cavoatrial junction was centrifugal, propagating superior to inferior at both the RA septum and RA free wall with collision at the CTI. When the RA and LA LAT mappings were combined, the activation and propagation revealed a biatrial macro–re-entrant circuit from the anterior LA to the superior cavoatrial junction in the RA likely via Bachmann’s bundle (BB), down to the inferior RA septum and then back to the LA on the lower septum; it then looped around the MA, back to the anterior LA **(Figure 3A)**. Entrainment in the right superior cavoatrial junction, RA septum, and MA showed they were all in the circuit with a PPI-TCL of <+20 ms. Ablation lesions were delivered at the earliest activation site in the RA at the superior RA septum and the AFL terminated **(Figures 3B and 3C)**. The patient remained in sinus rhythm during follow-up. An echo repeated 6 months later showed that the LVEF had normalized.

This study was approved by an investigational review board of the University of Florida.

## Discussion

Single-loop biatrial flutter is an uncommon form of atypical AFL, reported to be 0.5%–2.1% in patients with atypical AFL.^1,2^ Mikhaylov et al. reported 4 cases of clockwise peri-mitral AFL that changed into biatrial flutter with the same clockwise activation around the MA after anterior line ablation.^1^ Di et al. reported that a counterclockwise biatrial flutter was induced during the same procedure after a clockwise peri-mitral AFL was terminated by anterior line ablation.^3^ Our case shows that a counterclockwise peri-mitral flutter can also change into a counterclockwise biatrial flutter with anterior line ablation.

High-resolution LAT and bipolar voltage mapping combined with targeted entrainment are critical to delineate the re-entrant circuit of atypical AFL, which can possibly be composed of an inner loop, an isthmus, an outer loop, and adjacent and remote bystanders. It is important to identify which atrium or which part of a specific atrium is actively involved in the re-entrant circuit or is passively activated as a bystander.^4^ We only identified a single loop without the outer loop in this case. The right septum and peri-mitral LA were in the re-entrant circuit inner loop, while the lower right septum close to the CTI and the whole free wall of the RA were passively activated as bystanders during the biatrial flutter.

The electrical connection in between the RA and the LA is normally very robust, with both endo- and epicardial insertions based on anatomy and mapping.^5,6^ It includes BB, the septopulmonary bundle, the CS, and anterosuperior and posteroinferior interatrial connections at the septum. The BB is a parallelly aligned bundle of myocardial strands that stretch across the anterior interatrial groove and is the main pathway of interatrial conduction between the RA and LA.^5–7^ These interatrial connections will also participate in the re-entrant circuit of the biatrial flutter. In this case, the activation entered the RA from the LA via BB, with the earliest activation in the RA just below the SVC.

Either the insertion sites in the atria or the isthmus of the biatrial flutter can be targeted for ablation. Ablation on BB may cause bidirectional block of BB, resulting in LA activation changes during sinus rhythm.^8^ In patients with extensive LA scaring secondary to prior ablation or another atriopathy, there may not be intact RA–LA electrical coupling via the CS and septum. In this case, an ablation-induced bidirectional block of BB may cause disruption of the electrical connection between the RA and LA during sinus rhythm or even LAA isolation.^9^ The isthmus of the biatrial flutter can also possibly be targeted in the RA or LA, such as by lateral mitral isthmus linear ablation^3^; the CS close to the mitral isthmus^9^; or other LA or RA sites, including the CTI.^2^ Lateral line ablation can make it more difficult to achieve termination of AFL with due to epicardial connections.^10^

## Conclusion

Anterior line ablation can change a peri-mitral AFL into a biatrial flutter during ablation delivery or cause a future biatrial flutter. BB, CS, and septal connections can all be either the entrance site into or the exit site from the RA in the biatrial re-entrant circuit. High-resolution LAT mapping and bipolar voltage mapping combined with targeted entrainment are needed to identify the re-entrant circuit. RA mapping should be considered if the left AFL changes into a different AFL with a longer TCL during ablation, especially if the LAT range in the LA is much shorter than the TCL and/or with interruption of the activation sequence. Ablation at the entrance or exit site in either the RA or LA or ablation of the isthmus of the re-entrant circuit are options for ablation to terminate biatrial flutter.
